# Acute Ethanol Causes Hepatic Mitochondrial Depolarization in Mice: Role of Ethanol Metabolism

**DOI:** 10.1371/journal.pone.0091308

**Published:** 2014-03-11

**Authors:** Zhi Zhong, Venkat K. Ramshesh, Hasibur Rehman, Qinlong Liu, Tom P. Theruvath, Yasodha Krishnasamy, John J. Lemasters

**Affiliations:** 1 Department of Drug Discovery & Biomedical Sciences, Medical University of South Carolina, Charleston, South Carolina, United States of America; 2 Department of Biochemistry & Molecular Biology, Medical University of South Carolina, Charleston, South Carolina, United States of America; 3 Hollings Cancer Center, Medical University of South Carolina, Charleston, South Carolina, United States of America; University of California, Merced, United States of America

## Abstract

**Background/Aims:**

An increase of ethanol metabolism and hepatic mitochondrial respiration occurs *in vivo* after a single binge of alcohol. Here, our aim was to determine how ethanol intake affects hepatic mitochondrial polarization status *in vivo* in relation to ethanol metabolism and steatosis.

**Methods:**

Hepatic mitochondrial polarization, permeability transition (MPT), and reduce pyridine nucleotides, and steatosis in mice were monitored by intravital confocal/multiphoton microscopy of the fluorescence of rhodamine 123 (Rh123), calcein, NAD(P)H, and BODIPY493/503, respectively, after gavage with ethanol (1–6 g/kg).

**Results:**

Mitochondria depolarized in an all-or-nothing fashion in individual hepatocytes as early as 1 h after alcohol. Depolarization was dose- and time-dependent, peaked after 6 to 12 h and maximally affected 94% of hepatocytes. This mitochondrial depolarization was not due to onset of the MPT. After 24 h, mitochondria of most hepatocytes recovered normal polarization and were indistinguishable from untreated after 7 days. Cell death monitored by propidium iodide staining, histology and terminal deoxynucleotidyl transferase dUTP nick end labeling (TUNEL) was low throughout. After alcohol, mitochondrial NAD(P)H autofluorescence increased and decreased, respectively, in hepatocytes with polarized and depolarized mitochondria. Ethanol also caused steatosis mainly in hepatocytes with depolarized mitochondria. Depolarization was linked to ethanol metabolism, since deficiency of alcohol dehydrogenase and cytochrome-P450 2E1 (CYP2E1), the major ethanol-metabolizing enzymes, decreased mitochondrial depolarization by ∼70% and ∼20%, respectively. Activation of aldehyde dehydrogenase decreased depolarization, whereas inhibition of aldehyde dehydrogenase enhanced depolarization. Activation of aldehyde dehydrogenase also markedly decreased steatosis.

**Conclusions:**

Acute ethanol causes reversible hepatic mitochondrial depolarization *in vivo* that may contribute to steatosis and increased mitochondrial respiration. Onset of this mitochondrial depolarization is linked, at least in part, to metabolism of ethanol to acetaldehyde.

## Introduction

Alcoholic liver disease (ALD) remains the most common cause of liver-related mortality in the U.S. [1]. Despite extensive studies, mechanisms underlying ethanol damage are far from clear. Alcohol exposure causes a substantial increase of hepatic alcohol metabolism and oxygen consumption, a phenomenon named swift increase in alcohol metabolism (SIAM) [2]–[4]. High demand for oxygen after this respiratory burst may lead to pericentral (centrolobular) hypoxia [5], [6]. Although increased respiration should theoretically increase ATP generation by oxidative phosphorylation, alcohol treatment actually decreases hepatic ATP [7]–[9]. Furthermore, inhibition of mitochondrial fatty acid β-oxidation leads to rapid accumulation of neutral lipids within hepatocytes [10]. The respiratory burst of SIAM may be, in part, an adaptive response to oxidize the toxic metabolite acetaldehyde more rapidly and to increase NAD^+^ supply for alcohol metabolism.

The pioneering studies of Thurman's group showed that SIAM occurs only after *in vivo* treatment with alcohol [3], [4], [11], [12]. Livers isolated from ethanol-pretreated rats and mice show increased ethanol metabolism and oxygen consumption. However, infusion of ethanol into isolated livers from naive, untreated rodents does not cause enhanced ethanol metabolism or oxygen uptake. Enhanced ethanol metabolism and oxygen consumption after *in vivo* treatment was also confirmed *in vivo* in living animals [11]. The mechanism for SIAM remains unclear but most likely involves multiple factors, such as release of adrenergic hormones, prostaglandin E_2_ and inflammatory cytokines, increased gut permeability, endotoxemia, cross-talk between Kupffer cells and hepatocytes, and generation of H_2_O_2_ by peroxisomal β-oxidation [3], [13]–[15].

If oxidative phosphorylation remains intact after ethanol, then increased respiration implies an increase of ATP production and possibly an increase of mitochondrial membrane potential. Alternatively, if increased respiration is due to uncoupling, then a decrease of mitochondria potential with decreased mitochondrial ATP generation should occur. Since SIAM is an *in vivo* phenomenon, we sought to determine changes in hepatic mitochondrial polarization in living mice in response to ethanol. Intravital confocal/multiphoton microscopy provides a novel approach to visualize mitochondrial function in living animals [16], [17]. Here, we used this emerging technique to characterize alterations of mitochondrial function, cell death and steatosis after ethanol treatment. Our results show that acute ethanol induces reversible mitochondrial depolarization and steatosis that are linked to ethanol metabolism to acetaldehyde.

## Materials and Methods

### Animals and chemicals

Sources for animals and reagents are listed in Table 1.

**Table 1:** Sources for Animals and Chemicals.

*Items*	*Sources*
***Animals:***	
C57BL/6 mice	Jackson Laboratory, Bar Harbor, Maine
Cyp2E1^-/-^ mice	Dr. Frank Gonzales, National Cancer Institute
Deer mice	Peromyscus Genetic Stock Center, Columbia, SC
***Reagents:***	
Actin antibody	ICN, Costa Mesa, CA
Alcohol analytical kit	BioVision, San Francisco, CA
Alda-1	Dr. Daria Mochly-Rosen, Stanford University
ALT analytical kit	Pointe Scientific, Uncoln Park, MI
Aminobenzotriazole	Sigma-Aldrich, St. Louis, MO
BODIPY493/503	Invitrogen, Carlsbad, CA
Bromosulfophthalein	Sigma-Aldrich, St. Louis, MO
Calcein-AM	Biotium Inc., Hayward, CA
Chemiluminescence kit	Pierce Biotec., Rockford, IL
Disulfiram	Sigma-Aldrich, St. Louis, MO
Enliten ATP Assay System	Promega Corp., Madison, WI
MAA antibody	Dr. Todd Wyatt, Univ. of Nebraska Medical Center
4-HNE antibody	Alpha Diagnostics, Inc., San Antonio, TX
Polyethoxylated castor oil	Sigma-Aldrich, St. Louis, MO
Propidium iodide	Sigma-Aldrich, St. Louis, MO
Rhodamine 123	Sigma-Aldrich, St. Louis, MO
TMRM	Invitrogen, Carlsbad, CA
Triglyceride analytical kit	Enzymatic Standbio, Boerne, TX

Alda-1, *N*-(1,3-benzodioxol-5-ylmethyl)-2,6-dichlorobenzamide; ALT, alanine aminotransferase; calcein-AM, calcein acetoxymethyl ester, 4-HNE, 4-hydroxynonenal adducts; MAA, malondialdehyde-acetaldehyde adducts; TMRM, tetramethylrhodamine methylester.

### Animals and ethanol treatment

Male C57BL/6 mice (8–9 weeks), Cyp2E1–null mice, and alcohol dehydrogenase (ADH)-positive and ADH-negative deer mice had access to chow diet *ad libitum* before a single gavage with alcohol (1–6 g/kg) or vehicle (saline). Some wild-type mice were pretreated with a cytochrome P450 inhibitor, aminobenzotriazole (ABT, 100 mg/kg, *i.g.*), an aldehyde dehydrogenase (ALDH) inhibitor, disulfiram (DSF, 200 mg/kg, *i.p.*), or an ALDH activator, Alda-1 [18] (*N*-(1,3-benzodioxol-5-ylmethyl)-2,6-dichlorobenzamide, 50 mg/kg, *i.p.*) 30 min prior to ethanol or with cyclosporin A (CsA, 10 mg/kg, *i.g.*) at 1 h before ethanol. Vehicles for ABT, DSF, Alda-1 and CsA were saline, DMSO, DMSO and 8.3% polyethoxylated castor oil with 8.3% ethanol, respectively.

### Clinical chemistry and histology

Blood was collected 1–6 h after ethanol treatment. Alcohol and alanine aminotransferase (ALT) were measured using commercial analytical kits (Table 1) according to the manufacturers' protocols. Apoptosis was assessed by terminal deoxynucleotidyl transferase dUTP nick end labeling (TUNEL) [19]. Some liver tissue was frozen-sectioned and stained with Oil-Red-O staining to detect steatosis [20]. Paraffin sections after paraformaldehyde fixation were stained with periodic acid Schiff (PAS) to assess glycogen.

### Measurement of hepatic triglycerides

Liver tissue (200 mg) was homogenized in normal saline and extracted with 2∶1 chloroform/methanol. After centrifugation at 1700 rpm for 5 min, the chloroform phase was separated, dried in a speed vacuum centrifuge and resuspended in 1 ml of chloroform. Triglyceride was measured using an analytical kit (Table 1) [20].

### Hepatic ATP measurement

To assess the effects of ethanol on hepatic ATP, mice were treated with saline or ethanol (6 g/kg, *i.g*.), and livers were harvested 6 h later by freeze-clamping using aluminum tongs chilled in liquid nitrogen followed by storage at −80°C. ATP in trichloroacetic acid extracts was detected by luciferin-luciferase assay using an Enliten ATP Assay System (Table 1) [21], [22].

### Immunoblotting

Acetaldehyde is highly reactive, volatile and thus difficult to measure accurately. However, acetaldehyde rapidly reacts with other aldehydes/proteins to form adducts. Such adducts are potentially toxic to mitochondria and linked to ethanol hepatotoxicity [23]. Accordingly, malondialdehyde-acetaldehyde adducts (MAA), a hybrid acetaldehyde adduct [23]–[25], and 4-hydroxynonenal (4-HNE) adducts, an indicator of lipid peroxidation, were detected by immunoblotting, as described [20]. Immunoblotting was performed with primary antibodies specific for MAA, 4-HNE, and actin (Table 1) at 1∶1000, 1∶1000, and 1∶3000 dilutions, respectively, over night at 4°C. Horseradish peroxidase-conjugated secondary antibodies were applied, and detection was by chemiluminescence [20].

### Assay of hypoxia inducible factor (HIF)-1a mRNA by quantitative real-time PCR

Livers were harvested at 1 h and 6 h after ethanol treatment. HIF-1α mRNA was detected by quantitative real-time PCR, as described [26]. HIF-1α mRNA in liver was determined using a forward primer of 5′-GAAATGGCCCAGTGAGAAAA-3′ and a reverse primer of 5′-CTTCCACGTTGCTGACTTGA-3′. The abundance of mRNAs was normalized against hypoxanthine phospho-ribosyl-transferase (HPRT) using the ΔΔ*Ct* method.

### Intravital confocal and multiphoton microscopy

Intravital confocal and multiphoton microscopy was performed at 1 h to 7 days after saline or ethanol treatment, as described [16], [26]. Mice had access to chow diet and water ad libitum until imaging. Rhodamine 123 (Rh123) and tetramethylrhodamine methylester (TMRM), cationic fluorophores that are taken up by polarized mitochondria in response to the negative mitochondrial membrane potential, were used to monitor mitochondrial polarization after ethanol treatment. Propidium iodide (PI) and 4,4-difluoro-1,3,5,7,8-pentamethyl-4-bora-3a,4a-diaza-*s*-indacene (BODIPY493/503) were used to label nuclei of non-viable cells and fat droplets, respectively. Endogenous mitochondrial NAD(P)H was detected by blue autofluorescence. Onset of inner membrane permeabilization characteristic of the mitochondrial permeability transition (MPT) was detected after portal infusion of calcein acetoxymethyl ester (calcein-AM), which is cleaved by intracellular esterases to release calcein free acid into the cytosol, as described [27]. Under normal conditions calcein fluoresces in the cytosol, revealing mitochondria as dark voids due to the impermeability of mitochondrial membranes to calcein. These dark voids disappear after onset of the MPT as calcein enters the mitochondrial matrix space through MPT pores [16], [26], [27] [28] [22].

Under pentobarbital anesthesia (80 mg/kg, *i.p*.), a tracheotomy was performed, and an intravenous catheter (20 gauge) was inserted into the trachea, secured with a 5-0 silk suture, and connected to a small animal ventilator. The carotid artery was then cannulated with polyethylene (PE10) tubing. Rh123 (2 µmol/mouse), TMRM (1 µmol/mouse), PI (0.04 µmol/mouse), and/or BODIPY493/503 (25 µg/mouse) were infused singly or in combination in 0.4 mL of normal saline via the PE10 tubing over 10 min. Calcein-AM (1 mg/mouse) was injected slowly into the rectal vein. Bromosulfophthalein (6.6 µmol/mouse), an anion channel inhibitor, was injected into the rectal vein 5 min before calcein-AM to prevent biliary excretion of calcein. Imaging was started within 10 min after fluorophore infusion. Direct infusion of the fluorophores into the blood stream eliminated the potential influence of ethanol on absorption rates for the fluorophores and allowed rapid distribution of fluorophores into organs and cells.

After fluorophore loading, the abdomen was opened transversely to expose the liver. Laparotomized mice were then placed prone on the stage of a Zeiss LSM 510 NLO laser scanning confocal/multiphoton microscope. The liver was gently positioned over a coverslip and imaged with 25x and 63x water-immersion objective lenses. Settings for simultaneous imaging of Rh123 (green) plus PI (red), Rh123 (green) plus NAD(P)H autofluorescence (blue), TMRM (red) plus BODIPY493/503 (green), TMRM (red) alone or calcein (green) alone are listed in Table 2. During image acquisition, the respirator was turned off during ∼8 sec image scans to eliminate breathing movement artifacts. In preliminary studies, we observed that the preparations were stable for at least 1 h, but for the experiments presented imaging was completed within 30 min of fluorophore loading.

**Table 2:** Intravital Confocal/Multiphoton Microscopy Settings.

*Fluorophores*	*Imaging Mode*	*Excitation*	*Emission Filters*	*Color*
Rh123+PI	Two-photon	800 nm	500–550 & 650–710 nm	Green; red
Rh123+NAD(P)H	Two-photon	720 nm	500–550 & 435–485 nm	Green; blue
Calcein	Two-photon	720 nm	500–550 nm	Green
TMRM± BODIPY493/503	Single-photon	543&488 nm	565–615 & 500–530 nm	Red; green

Calcein-AM (1 mg/mouse); Rh123, rhodamine 123 (2 µmol/mouse); PI, propidium iodide (0.04 µmol/mouse); TMRM, tetramethylrhodamine methylester (0.8 µmol/mouse); BODIPY493/503 (25 µg/mouse).

To quantify labeling, 10 or more images were collected randomly from the liver of each mouse. Hepatocytes in these fields (∼250 cells) were scored in a blinded fashion for bright punctate Rh123 or TMRM fluorescence representing cells with polarized mitochondria versus a dimmer diffuse cytosolic fluorescence representing cells with depolarized mitochondria. For individual hepatocytes, ethanol caused mitochondrial depolarization almost always in an all-or-none manner. Thus, the distinction between punctate and diffuse staining was generally unambiguous. Rarely, mitochondria were not all depolarized within a single hepatocyte, and we scored such cells based on whether the majority of mitochondria had lost punctate labeling. Nonviable PI positive cells, indicated by red nuclear fluorescence, were also counted.

In some mice, simultaneous imaging of BODIPY493/503-labeled fat droplets (green) and mitochondrial polarization by TMRM (red) was performed at 2 h after ethanol treatment (6 g/kg, *i.g.*). The number of fat droplets in hepatocytes with and without mitochondrial depolarization was assessed in a blinded manner in 10 random fields per liver.

Reduced pyridine nucleotides (NADH plus NADPH) fluoresce blue, and previous studies showed that nearly all NAD(P)H autofluorescence arises from mitochondria, whereas cytosolic NAD(P)H fluorescence is highly quenched [29], [30]. Mitochondrial NADPH/NADP is in dynamic equilibrium with mitochondrial NADH/NAD via the electrogenic mitochondrial transhydrogenase. Blue autofluorescence in liver represents mitochondrial NAD(P)H regardless of whether autofluorescence is measured from whole liver by whole organ fluorometry, from sublobular locations with microlight guides, or from single cells/mitochondria by confocal/multiphoton microscopy [29]–[31]. Reduced pyridine nucleotides are relatively fragile and readily undergo photooxidation and photodamage. To minimize such changes, we used a low laser power setting that yielded images with lower signal-to-noise characteristics than for our other imaging.

### Measurement of serum Rh123

At 6 h after gavage with ethanol (6 g/kg) or an equal volume of saline, Rh123 was infused into the carotid artery under anesthesia as described above, and blood was collected from the vena cava immediately and after 10 and 30 min. Serum was obtained by centrifugation at 12,000 rpm for 10 min and stored in the dark at −80°C. After thawing, serum was diluted 10-fold in saline, and fluorescence was measured using excitation and emission wavelengths of 511 and 534 nm, respectively, with a Spectra Max M2 plate reader (Molecular Devices, Sunnyvale, CA) in comparison to Rh123 standards.

### Hepatic ischemia/reperfusion in mice

The effect of cyclosporin A (CsA) on the MPT was tested in a hepatic ischemia/reperfusion (I/R) model as a positive control for its effectiveness *in vivo* since CsA is a classical MPT inhibitor. Our previous studies demonstrated that hepatic I/R causes the MPT onset *in vivo*, leading to mitochondrial depolarization [16], [32]. At 1 h after vehicle- and CsA (10 mg/kg, i.g.) treatment, hepatic ischemia was induced by clamping the artery and portal vein to the upper three lobes of the liver (*i.e.*, about 70% of total liver). The ischemic liver was reperfused by opening the vascular clamp 1 h later as described previously [16]. Mitochondrial polarization status was detected by intravital multiphoton microscopy of Rh123 at 2 h after reperfusion.

### Statistical analysis

All groups were compared using ANOVA plus Student-Newman-Keuls' post-hoc test or Student's t-test, as appropriate. Values are means ± SEM. Differences were considered significant at p<0.05.

### Ethics Statement

All animals were given humane care in compliance with institutional guidelines using protocols approved by the Institutional Animal Care and Use Committee of the Medical University of South Carolina. All surgery was performed under sodium pentobarbital anesthesia (80 mg/kg, *i.p*.).

## Results

### Ethanol caused mitochondrial depolarization *in vivo*

**Figure 1:**
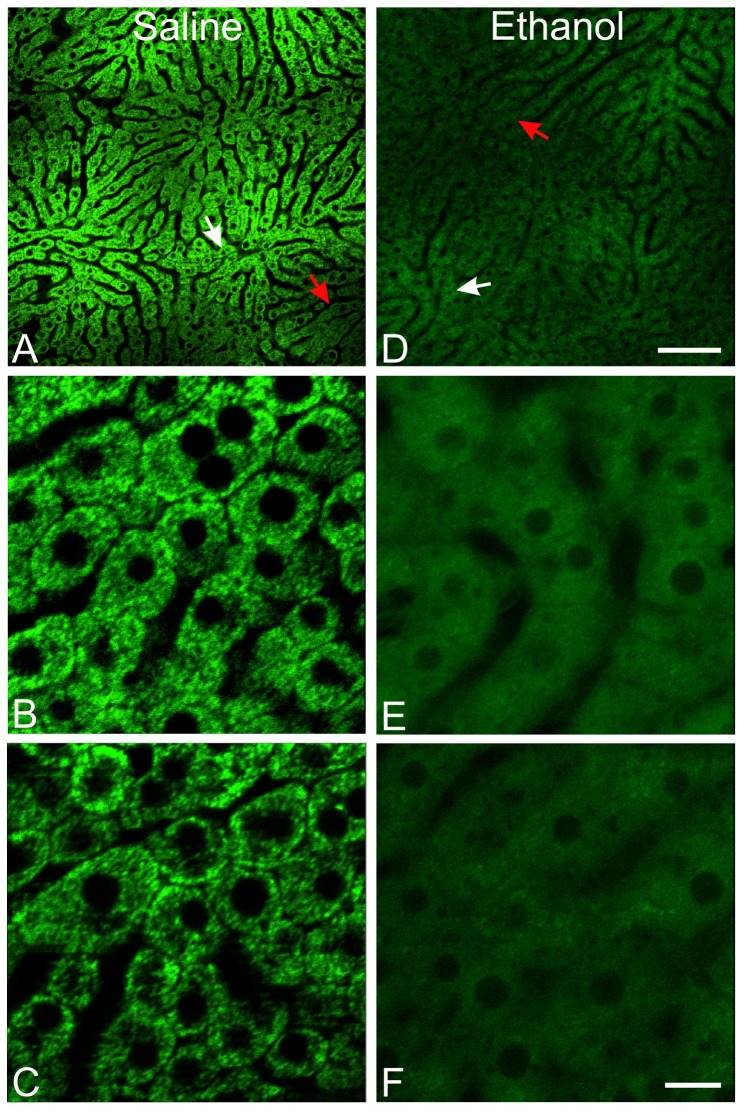
Acute ethanol causes widespread mitochondrial depolarization in the liver. Mice were treated with saline (left column) or ethanol (6 g/kg, *i.g*., right column). Intravital multiphoton microscopy of Rh123 was performed after 6 h using a 25× water objective lens. **A** and **D**, zooming at 0.7×. **B**, **C**, **E** and **F**, zooming at 4×. White arrows identify zooming areas shown in **B** and **E**. Red arrows identify zooming areas shown in **C** and **F**. Darker areas are pericentral regions of the liver lobules. Bar is 100 µm in A and D and 10 µm in B, C, E and F.

Using a 25x objective lens, portions of several liver lobules could be observed at the same time (Fig. 1A,D). Selective zooming showed that mitochondria of hepatocytes in different regions of livers of control mice were all polarized (Fig. 1B,C). In marked contrast, ethanol (6 g/kg) caused widespread mitochondrial depolarization which was not specific to a particular hepatic zone (Fig. 1E,F).

**Figure 2:**
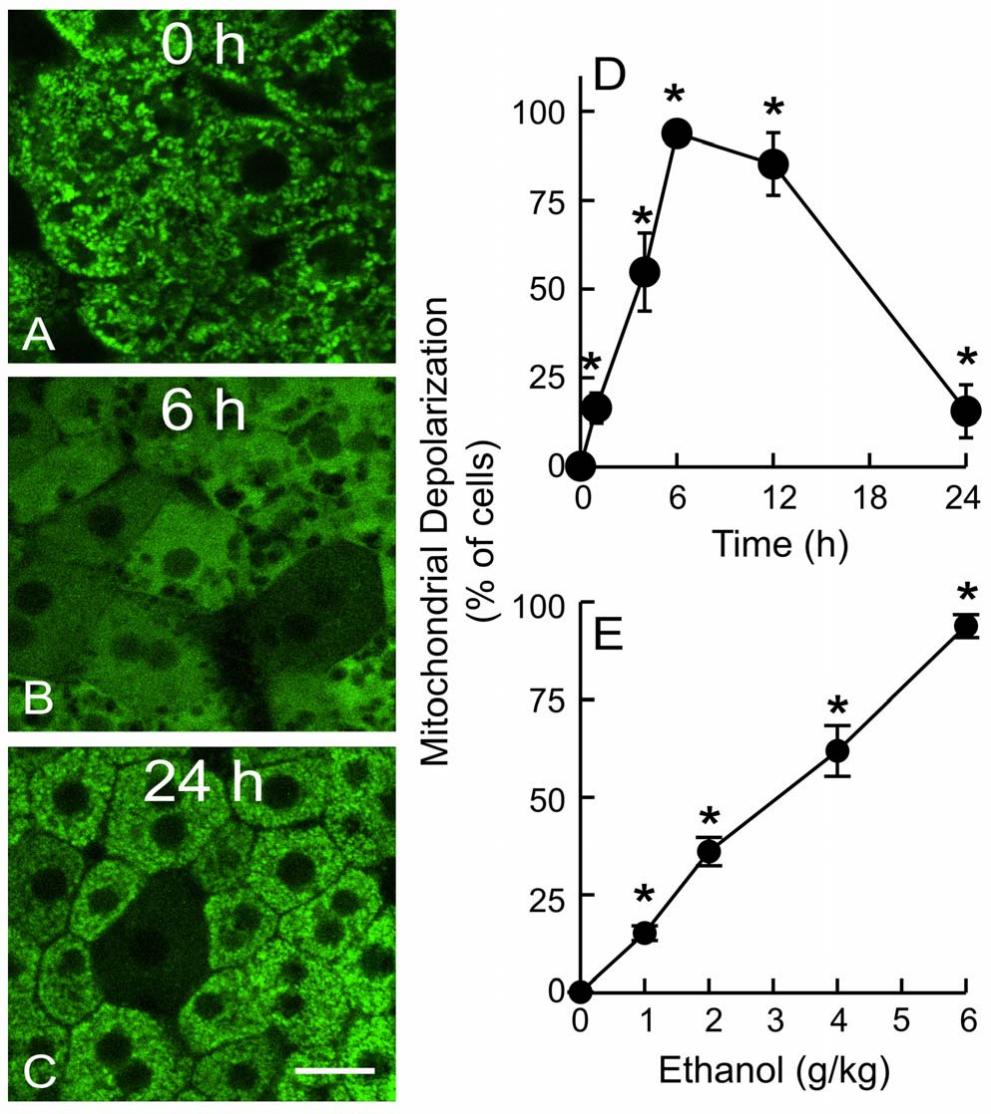
Ethanol causes reversible mitochondrial depolarization in vivo in a dose-dependent manner. Mice were gavaged with one dose of ethanol (0–6 g/kg) in saline, and mitochondrial polarization was detected by intravital multiphoton microscopy of Rh123 at 0 to 24 h after treatment. Representative images from mice treated with 6 g/kg ethanol are shown in **A**–**C**. Bar is 10 µm. **D**: time course of mitochondrial depolarization after treatment with 6 g/kg ethanol; **E**: dose-dependency of mitochondrial depolarization at 6 h after ethanol treatment. Values are means ± SEM (n = 4–5 per group). *, p<0.05 vs no ethanol.

The extent of depolarization was quantified by capturing 10 or more random images per liver at high magnification (63× objective lens). In saline-treated mice, green Rh123 fluorescence was punctate in virtually every hepatocyte, indicating mitochondrial polarization, and red PI labeling of nuclei was very rare (Fig. 2A). After ethanol treatment (6 g/kg), mitochondria of individual hepatocytes began to depolarize within 1 h (∼16% cells, p<0.05 vs saline; Fig. 2D). For individual hepatocytes, ethanol caused mitochondrial depolarization almost always in an all-or-none manner. Over time, the number of hepatocytes with depolarized mitochondria increased and peaked after 6 h, at which point ∼94% of hepatocytes contained depolarized mitochondria (Fig. 2B,D). Mitochondrial depolarization remained high (∼85%) at 12 h, decreased to ∼15% after 24 h (Fig. 2C,D) and was indistinguishable from the saline-treated group after 7 days (not shown). Cell death revealed by PI staining was rare during this 24 h period. These data indicated that acute ethanol caused widespread, reversible mitochondrial depolarization *in vivo* over a period of 1 to 24 h. Ethanol-induced mitochondrial depolarization was an *in vivo* phenomenon and does not occur *in vitro*. Specifically, ethanol treatment of cultured hepatocytes *in vitro* does not affect their ability to accumulate cationic fluorophores into their mitochondria, namely that the mitochondria are not depolarized by ethanol, as shown previously [74]–[76]. We repeated these experiments to show that mitochondria of cultured rat hepatocytes in growth medium continued to retain membrane potential-indicating TMRM (300 nM) even after exposure to 100 mM ethanol for 3 h (data not shown).

### Loss of mitochondrial Rh123 fluorescence after ethanol was unlikely due to upregulation of organic cation transporters or changes in Rh123 pharmacokinetics

Ethanol-dependent changes of biliary cation transporters, such as multidrug resistance-1 (MDR1), MDR2 and others, might influence intravital detection of mitochondrial polarization by Rh123. Fluorescence of fluorophores excreted into bile canaliculi (e.g. biliary excretion of ester-loaded anionic fluorophores) is readily observable in a characteristic “chicken wire” pattern (not shown). However, we did not observe biliary excretion of cationic Rh123 in either ethanol-treated or untreated mice within the time frames of imaging (Fig. 2A,B).

**Figure 3:**
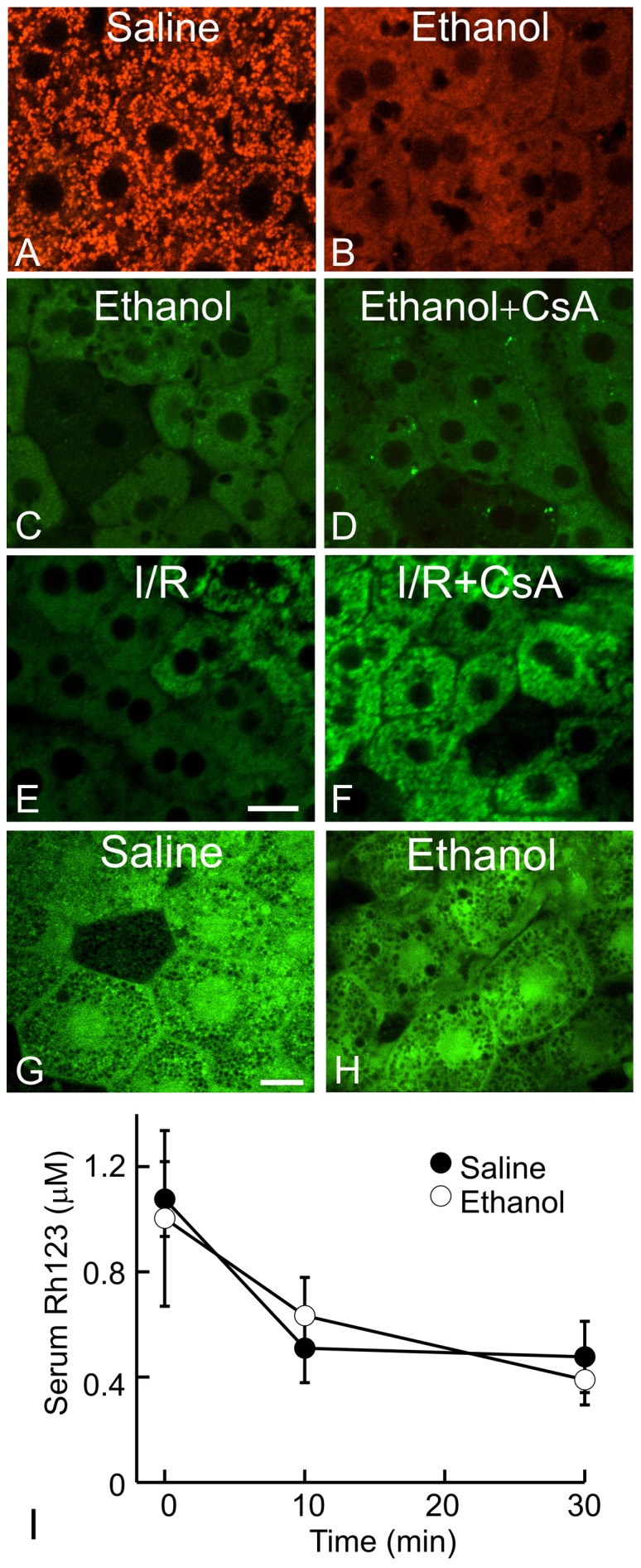
Loss of mitochondrial Rh123 fluorescence after ethanol is unlikely due to upregulation of organic cation transporters, the mitochondrial permeability transition or changes in Rh123 pharmacokinetics. In A–D, G and H, mice were treated with saline or ethanol (6 g/kg, *i.g*.), and images were collected at 6 h after treatment. In A and B, TMRM fluorescence was detected by intravital confocal microscopy. In C, D, E and F, mice were given vehicle or cyclosporin A (CsA, 10 mg/kg, *i.g.*). In E and F, sham-operation and hepatic ischemia/reperfusion (I/R) was performed, and images were collected 2 h later. In C, D, E and F, intravital multiphoton microscopy of Rh123 was performed to detect mitochondrial depolarization. In G and H, calcein fluorescence was imaged. Representative images of 3–4 mice per group are shown. Bars are 10 and 5 µm in E and G, respectively. In I, Rh123 was infused 6 h after saline or ethanol treatment. Blood was collected at 0, 10 and 30 min after Rh123 loading, and serum Rh123 was measured. Serum Rh123 was not significantly different between saline and ethanol-treated mice (n = 3 per group).

Mitochondrial uptake of TMRM, a more membrane-permeant cationic fluorophore than Rh123 whose equilibrium distribution in response to membrane potential should be less influenced by cation transporters [33], showed identical ethanol-induced changes as with Rh123; namely, mitochondrial TMRM fluorescence was lost in an all-or-none fashion from individual hepatocytes after ethanol treatment (Fig. 3A,B).

CsA is a potent inhibitor of biliary cation transport that blocks MDR-dependent Rh123 excretion [34], [35]. CsA had no effect on Rh123 release after ethanol, further confirming that Rh123 release was not due to MDR activity (Fig. 3C,D). CsA is also a potent blocker of the MPT. As a positive control, we tested the effect of CsA on *in vivo* MPT-dependent mitochondrial depolarization after hepatic ischemia-reperfusion (I/R) [16]. Our previous studies showed that mitochondrial depolarization after hepatic I/R is due to the MPT onset based on entry of calcein into mitochondria and inhibition by NIM811, another specific MPT inhibitor [16], [32]. After hepatic I/R, mitochondrial depolarization occurred in majority of hepatocytes as expected (Fig. 3E). CsA pretreatment markedly decreased mitochondrial depolarization (Fig. 3F). However, at the same dosage, CsA did not block mitochondrial depolarization after acute ethanol treatment (Fig. 3C,D). Moreover after ethanol treatment, calcein did not translocate from the cytosol into the matrix of depolarized mitochondria (Fig. 3G and H), as occurs after CsA-sensitive MPT onset following I/R and bile duct ligation [16], [26]. Thus, ethanol-induced mitochondrial depolarization *in vivo* was not due to MPT onset.

Serum concentrations of Rh123 were measured to evaluate the effect of acute ethanol treatment on Rh123 pharmacokinetics. Just after infusion, serum Rh123 in control mice increased to 1.1 µM, which declined gradually to 0.48 µM after 30 min, our time frame for imaging (Fig. 3I). Ethanol treatment did not alter serum Rh123 concentrations in comparison to the control mice within this time frame (Fig. 3I).

### Dose-dependency of ethanol-induced mitochondrial depolarization

As little as 1 g/kg of ethanol induced mitochondrial depolarization in ∼15% of hepatocytes (p<0.05 vs. saline, Fig. 2E), a dose leading to a blood alcohol of ∼90 mg/dL after 1 h. As the ethanol dose increased, mitochondrial depolarization progressively increased to a maximum of 94% of hepatocytes after 6 g/kg (Fig. 2E), a dose leading to a blood alcohol of ∼230 mg/dL after 1 h. These results showed that ethanol caused mitochondrial depolarization in a dose-dependent manner and at pharmacologically relevant blood alcohol levels.

### Ethanol-induced mitochondrial depolarization was not caused by anoxia but was consistent with uncoupling

**Figure 4:**
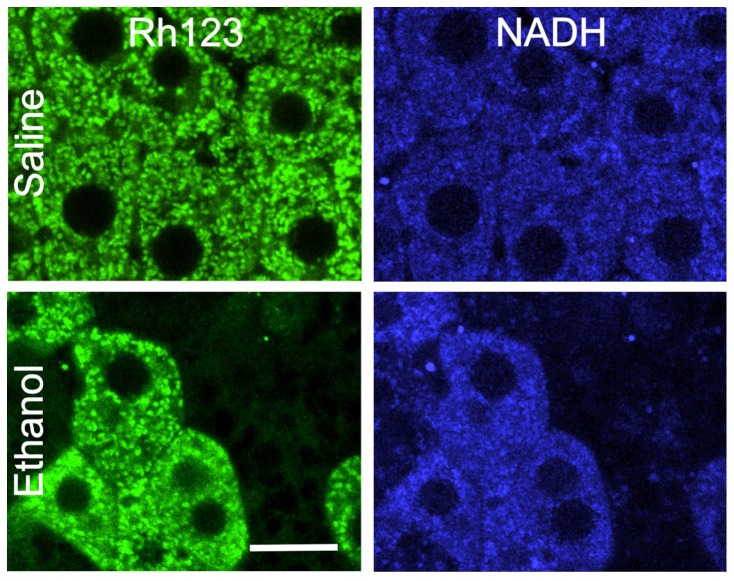
Ethanol-induced mitochondrial depolarization is associated with oxidation of mitochondrial NAD(P)H. Mice were gavaged with saline or ethanol (6 g/kg). Mitochondrial polarization was detected by Rh123 fluorescence (left column), and mitochondrial NAD(P)H (right column) was detected by autofluorescence after 2 h. Representative images of 4 mice per group are shown. Bar is 10 µm.

To determine whether mitochondrial depolarization after ethanol was due to anoxia or uncoupling *in vivo*, we imaged mitochondrial NAD(P)H autofluorescence. NAD(P)H is fluorescent whereas NAD(P)^+^ is not. Previously, fluorescence of reduced pyridine nucleotides (NADH and NADPH) in the cytosol of liver was shown to be highly quenched and that nearly all NAD(P)H fluorescence arises from mitochondria [29], [30]. Numerous studies have used NAD(P)H autofluoresence to study mitochondrial function [29], [30], [36], [37]. In saline-treated livers, blue punctate NAD(P)H fluorescence was observed, consistent with mitochondrial localization (Fig. 4). NAD(P)H fluorescence was also observed in the absence of Rh123 infusion (not shown), indicating that blue fluorescence was not bleed-through of Rh123 fluorescence. At 2 h after ethanol (6 g/kg) when mitochondria of ∼40% of hepatocytes were depolarized, mitochondrial NAD(P)H fluorescence in hepatocytes with polarized mitochondria increased relative to saline-treated livers, consistent with NADH formation by ethanol metabolism. However in hepatocytes with depolarized mitochondria after ethanol treatment, mitochondrial NAD(P)H fluorescence was decreased compared to adjacent hepatocytes with polarized mitochondria (Fig. 4). Mitochondrial depolarization with oxidation of NAD(P)H is consistent with mitochondrial uncoupling but inconsistent with anoxia, since in anoxia mitochondrial NAD(P)H increases maximally. Because reduced pyridine nucleotides are relatively fragile and readily undergo photooxidation and photodamage, we used a low laser power setting to minimize photodamage in these experiments. Consequently, NAD(P)H images displayed a poorer signal-to-noise characteristic than our other imaging. Nonetheless, integration of total cellular autofluorescence from these noisy images accurately reflected total mitochondrial pyridine nucleotide fluorescence within the cells even if individual mitochondria were not always readily identifiable.

HIF-1α mRNA was detected at 1 h and 6 h after ethanol treatment. Although mitochondrial depolarization occurred as early as 1 h after ethanol treatment, HIF-1α mRNA was not increased after 1 h. After 6 h, HIF-1α mRNA began to show a trend to increase (2.8-fold, p = 0.067). These data suggested that stimulation of respiration by uncoupling after ethanol led to decreased oxygen concentration within the liver and HIF-1α signaling, as reported previously [5], [6], [31].

### Alcohol-induced mitochondrial depolarization depended on ethanol metabolism by alcohol dehydrogenase and cytochrome P450

**Figure 5:**
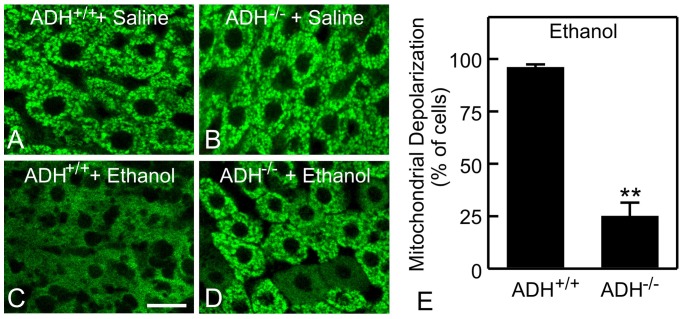
Role of alcohol dehydrogenase in ethanol-induced mitochondrial depolarization. ADH positive (ADH^+/+^) and negative (ADH^-/-^) deer mice were gavaged with saline (**A** and **B**) or ethanol (6 g/kg, **C** and **D**). Rh123 fluorescence was detected after 6 h. Representative images of 4 mice per group are shown. Bar is 10 µm. **E**: quantification of cells with mitochondrial depolarization at 6 h after ethanol treatment. Values are means ± SEM (n = 4 per group). **, p<0.01 vs ADH positive deer mice.

ADH is the major pathway of ethanol metabolism in liver. Mitochondria were polarized in ADH-positive and ADH-negative deer mice without ethanol treatment (Fig. 5A,B). After ethanol (6 g/kg), mitochondria depolarized in most hepatocytes of ADH-positive deer mice (96%) (Fig. 5C,E), as seen with C57BL/6 mice, but depolarization occurred in only 25% of hepatocytes of ADH-negative deer mice (p<0.01 vs ADH-positive, Fig. 5D,E), indicating the important role of ADH in mitochondrial depolarization.

**Figure 6:**
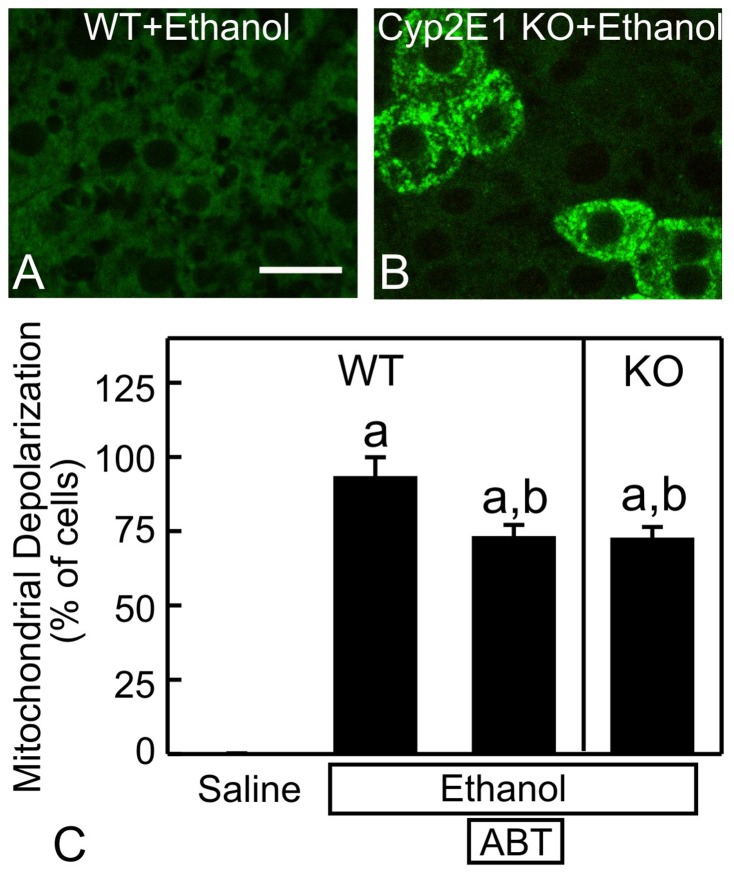
Role of cytochrome P450-dependent ethanol metabolism in mitochondrial depolarization. Wild-type (WT) (**A**) and Cyp2E1 knock-out (KO) (**B**) mice were gavaged with ethanol (6 g/kg). Rh123 fluorescence was detected after 6 h. Some WT mice were pretreated with aminobenzotriazole (ABT, 100 mg/kg, *i.p.*), a cytochrome P450 inhibitor, 30 min prior to ethanol. In **C**, mitochondrial depolarization as a percentage of all hepatocytes is plotted. Values are means ± SEM (n = 4 per group). a, p<0.05 vs WT with saline; b, p<0.05 vs WT with ethanol. Bars are 10 µm.

The cytochrome P450-dependent microsomal ethanol-oxidizing system (MEOS) is also involved in ethanol metabolism. Ethanol caused widespread mitochondrial depolarization in wild-type mice, as expected (Fig. 6A). Pretreatment with 100 mg/kg ABT, a cytochrome-P450 inhibitor, decreased mitochondrial depolarization by ∼20% (p<0.05, Fig. 6C). Depolarization did not decrease further at higher doses of ABT (not shown). Cyp2E1 is the major subtype of cytochrome P450 responsible for ethanol metabolism [38]. In Cyp2E1-null mice, ethanol-induced mitochondrial depolarization also decreased by ∼20% (p<0.05, Fig. 6B,C), consistent with data after cytochrome P450 inhibition.

### Role of aldehyde dehydrogenase in alcohol-induced mitochondrial depolarization

**Figure 7:**
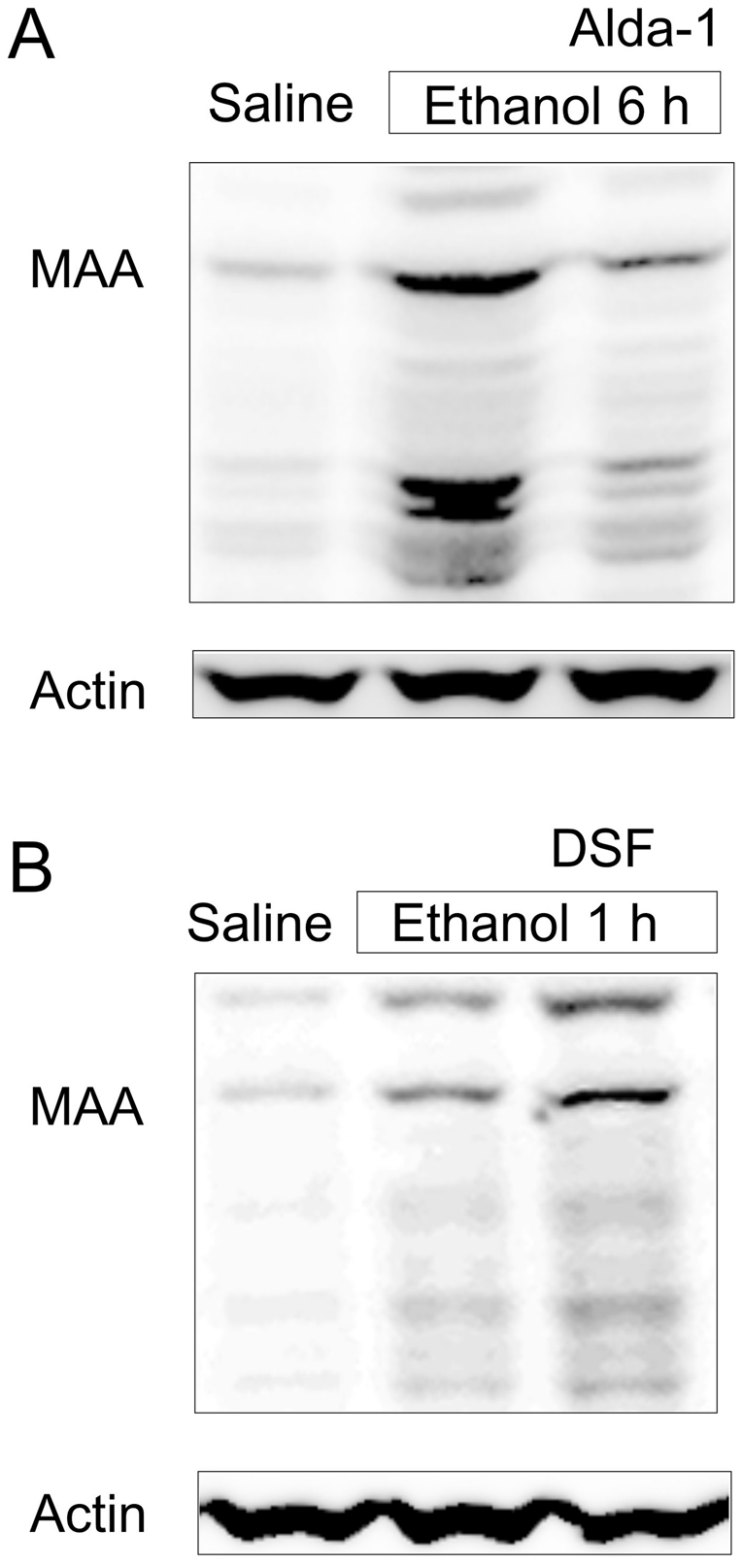
Alda-1 decreases whereas disulfiram increases malondialdehyde-acetaldehyde adducts after ethanol treatment. Mice were injected with vehicle, Alda-1 (50 mg/kg, *i.p.*) or DSF (200 mg/kg, *i.p.*) 30 min before gavage of saline or ethanol (6 g/kg). MAA adducts were detected in liver tissue at 6 h after ethanol treatment (**A**) or at 1 h after ethanol (**B**) by immunoblotting. Shown are representative images of gels (n = 4 per group).

**Figure 8:**
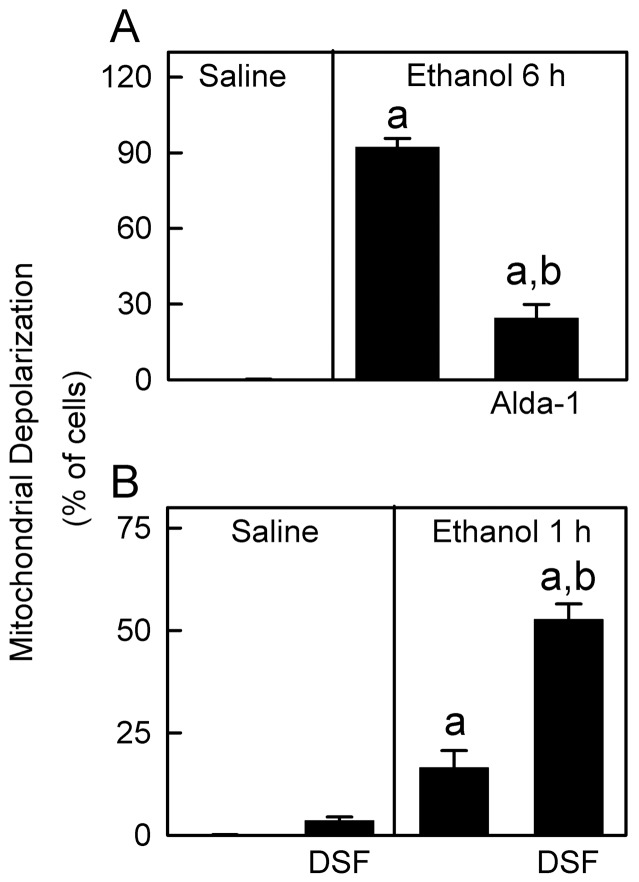
Role of aldehyde dehydrogenase in ethanol-induced mitochondrial depolarization. Mice were injected with vehicle, Alda-1 (50 mg/kg, *i.p.*) or DSF (200 mg/kg, *i.p.*) 30 min before gavage of saline or ethanol (6 g/kg), and Rh123 fluorescence was detected after 6 h (**A**) and 1 h (**B**), respectively. Values are means ± SEM (n = 4 per group). a, p<0.05 vs saline; b, p<0.05 vs ethanol without Alda-1 or DSF.

Ethanol oxidation by ADH and MEOS produces acetaldehyde, which is highly reactive and rapidly binds to other aldehydes/proteins to form adducts. MAA is a hybrid acetaldehyde adduct [23], [24], [25]. In the livers of saline-treated mice, only weak MAA-positive bands were detected (Fig. 7A). After ethanol treatment, multiple MAA-positive bands increased, indicating formation of MAA protein adducts. Acetaldehyde from ethanol oxidation is degraded by mitochondrial aldehyde dehydrogenase-2 (ALDH2). Increases in MAA after ethanol treatment were blunted by Alda-1, an ALDH2 activator [18] (Fig. 7A). Alda-1 also decreased mitochondrial depolarization after ethanol (6 g/kg) from 92% to 24% (p<0.01; Fig. 8A).

At 1 h after acute ethanol treatment, MAA adducts increased slightly in the liver, and DSF, which inhibits ALDH, increased the MAA adducts compared to ethanol alone (Fig. 7B). After saline, DSF did not cause a statistically significant change of mitochondrial depolarization (Fig. 8B). At 1 h after ethanol treatment without DSF, mitochondria depolarized in ∼16% of hepatocytes, which increased to 53% in the presence of DSF (Fig. 8B). These findings are consistent with the conclusion that acetaldehyde plays an important role in mitochondrial depolarization.

### Ethanol increases oxidative stress

**Figure 9:**
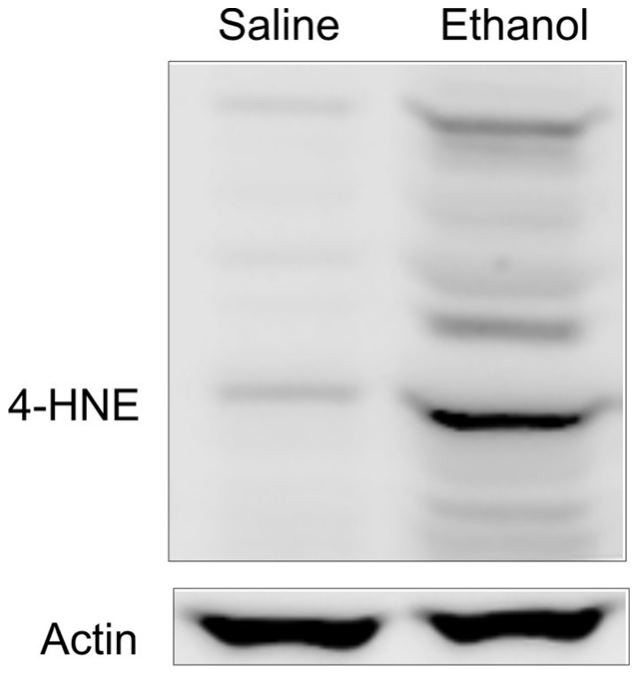
Ethanol increases 4-hydroxynonenal adduct formation. Mice were gavaged with saline or ethanol (6 g/kg). 4-HNE adducts were detected in liver tissue at 6 h after ethanol treatment by immunoblotting. Shown are representative images of gels (n = 3 per group).

Previous studies showed that ethanol increases oxidative stress and that reactive oxygen species may contribute to mitochondrial dysfunction [39]. Accordingly we measured 4-HNE, a product of lipid oxidation, at 6 h after ethanol treatment. Only weak 4-HNE-positive bands were detected without ethanol treatment (Fig. 9). After ethanol treatment, multiple 4-HNE-positive bands increased, indicating formation of 4-HNE protein adducts.

### Mitochondrial depolarization was associated with steatosis

**Figure 10:**
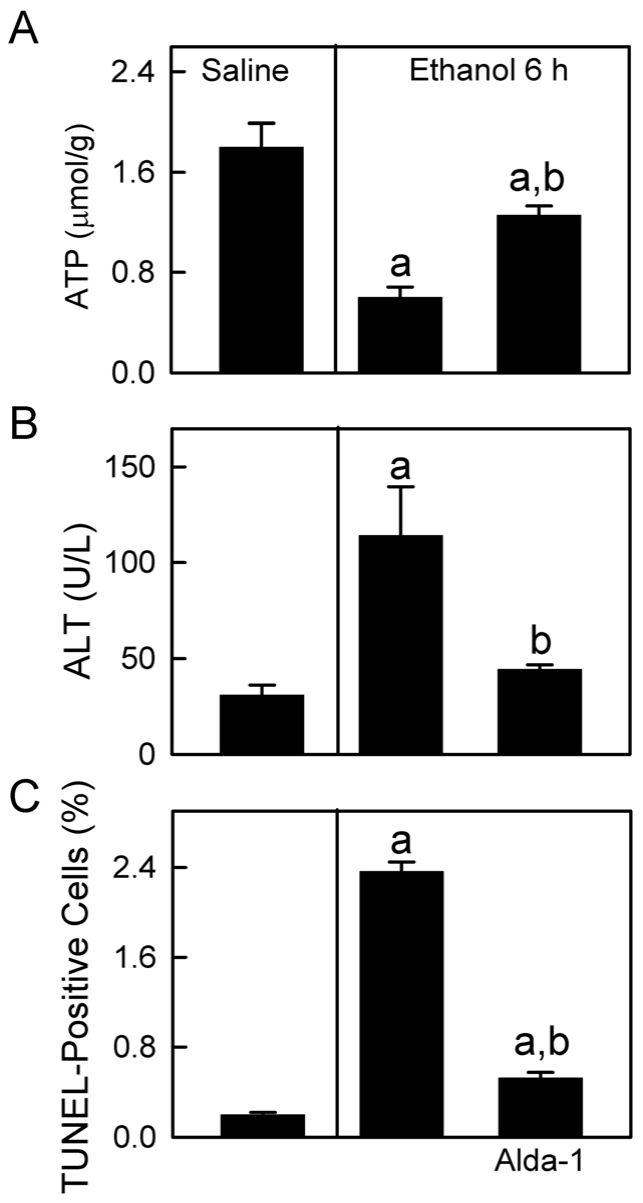
Ethanol decreases hepatic ATP and causes modest liver injury: protection by Alda-1. Mice were injected with vehicle or Alda-1 (50 mg/kg, *i.p.*) 30 min before gavage of saline or ethanol (6 g/kg), and blood and livers were collected 6 h later for hepatic ATP (**A**), serum ALT (**B**) and TUNEL (**C**) measurement. TUNEL-positive cells in liver sections were counted in 10 random fields. Values are means ± SEM (n = 4 per group). a, p<0.05 vs saline; b, p<0.05 vs ethanol without Alda-1.

**Figure 11:**
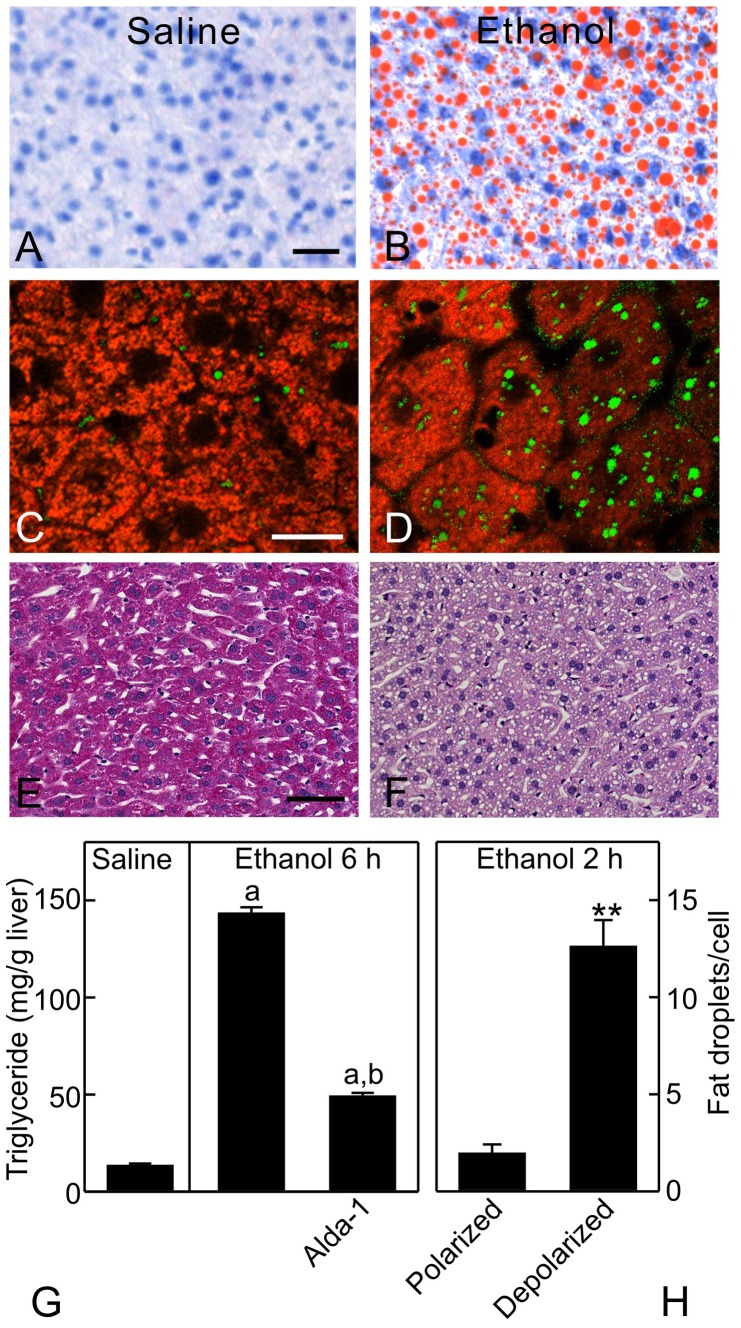
Ethanol causes steatosis in hepatocytes with depolarized mitochondria. Mice were gavaged with saline or ethanol (6 g/kg), and livers were collected 6 h later for Oil-Red-O (**A** and **B**; bar is 20 µm), PAS staining (**E** and **F**; bar is 50 µm), and triglyceride measurement (**G**). In **G**, some mice were injected with vehicle or Alda-1 (50 mg/kg, *i.p.*) 30 min before gavage of saline or ethanol (6 g/kg). Values are means ± SEM (n = 4 per group). a, p<0.05 vs saline; b, p<0.05 vs ethanol without Alda-1. Intravital confocal microscopy of TMRM (red) and BODIPY 493/503 (green) was performed after 2 h (**C** and **D**; bar is 10 µm). Representative images from 4 mice per group are shown. **H** shows the average number of fat droplets per cell in hepatocytes with polarized and depolarized mitochondria. **, p<0.01 compared to polarized (n = 4 per group).

After the largest dose of ethanol administered (6 g/kg), hepatic ATP decreased from 1.8 µmol/g liver to 0.6 µmol/g liver at 6 h (p<0.01, Fig. 10A). ALT increased from 31 to 114 U/L (Fig. 10B), and TUNEL-positive cells increased from 0.2% to 2.4% (Fig. 10C), indicating mild liver injury. Moreover, ethanol caused hepatic steatosis as indicated by markedly increased Oil-Red-O staining (Fig. 11C,D) and hepatic triglycerides (Fig. 11G). To observe the relation between mitochondrial depolarization and steatosis at the cellular level, we performed intravital confocal imaging of TMRM (red) and BODIPY493/503 (green) at 2 h after ethanol (6 g/kg). Mitochondria in ∼40% of hepatocytes were depolarized, as indicated by diffuse TMRM fluorescence (Fig. 11D, compare to C). Fat droplets labeled by BODIPY493/503 were 2 per hepatocyte with polarized mitochondria but increased to 13 in each hepatocyte with depolarized mitochondria (p<0.05, Fig. 11C,D,H). Alda-1 treatment, which activates ALDH2, restored ATP from 0.6 to 1.3 µmol/g liver and decreased serum ALT from 114 to 44 U/L, TUNEL-positive cells from 2.4% to 0.5% and hepatic triglyceride from 143 to 49 mg/g liver after ethanol treatment (Fig. 10 and Fig. 11G). Glycogen staining by PAS was distributed widely in livers of mice treated with saline but decreased substantially in livers of mice treated with ethanol (Fig. 11E,F), indicating depletion of glycogen.

## Discussion

### Acute ethanol causes hepatic mitochondrial dysfunction in living mice

One of the earliest manifestations of hepatocyte injury by alcohol is morphological and functional abnormalities of mitochondria [7], [40]. Chronic ethanol treatment selectively decreases mitochondrial glutathione [41]. Acetaldehyde forms protein adducts in mitochondria and other cellular structures of both human alcoholics and animals, which contributes to ethanol toxicity in organs [23]. Mitochondrial DNA (mtDNA) deletions and alterations in β-oxidation enzymes occur in alcoholic patients with hepatic steatosis and in animals after ethanol exposure [42], [43], suggesting that mtDNA and protein damage impairs mitochondrial β-oxidation of fatty acids. In some hands, exposure of cultured hepatocytes to ethanol causes MPT onset and apoptosis [44]. Overall, ethanol-induced mitochondrial alterations likely impair ATP production and thus may enhance hepatic susceptibility to injury from alcohol-induced centrolobular hypoxia [45]. In support of impaired ATP production, we found a 66% decrease of ATP after the largest dose of ethanol administered, and this effect was blunted by enhanced acetaldehyde degradation (Fig. 9A).

Despite extensive studies, the effects of ethanol on mitochondrial polarization status *in vivo* have not previously been determined. Detection of mitochondrial polarization in living animals has been difficult, but recent advances in intravital confocal/multiphoton microscopy make such *in vivo* monitoring now possible to permit non-destructive high resolution imaging deep into thick living specimens. Using multiphoton microscopy, we observed widespread hepatic mitochondrial depolarization in living mice after acute ethanol treatment. Ethanol-induced hepatic mitochondrial depolarization was rapid, reversible and dependent on the dose of ethanol (Fig. 2). This finding is novel in that no previous study describes such an *in vivo* pattern of reversible mitochondrial depolarization in the livers of mice after acute ethanol treatment.

Reversible mitochondrial depolarization after acute ethanol treatment was not due to MPT onset, since CsA did not prevent depolarization and since inner membrane permeabilization did not occur as shown by the absence of calcein movement into the mitochondrial matrix (Fig. 3). Mitochondria isolated from rats chronically treated with ethanol have increased propensity for the MPT [46]. The MPT leads to large amplitude mitochondrial swelling, outer membrane rupture, release of cytochrome *c* and cell death [47]. However, mitochondria isolated from the livers of ethanol-treated mice are structurally intact, indicating that the MPT had not yet occurred in these mitochondria [46], which is consistent with our findings that acute ethanol did not cause MPT onset *in vivo* and earlier findings by others that ethanol causes only mild killing (<10%) of isolated rat hepatocytes [9]. Rather, increased propensity for the MPT in mitochondria of livers of ethanol-treated animals may produce vulnerability to subsequent “hits” in the multi-hit pathogenesis of alcoholic liver disease.

Ethanol-induced loss of mitochondrial Rh123 fluorescence was also not due to an effect of ethanol on the pharmacokinetics of Rh123. Potential-indicating Rh123 and TMRM were directly infused into the carotid artery, and blood concentrations Rh123 were similar in mice with and without ethanol treatment (Fig. 3). A direct effect of ethanol on Rh123 fluorescence is also unlikely, since adjacent hepatocytes often showed distinctly different staining patterns after ethanol treatment, but such adjacent cells will have been exposed to the same concentration of highly cell-permeant ethanol. Based on our use of these fluorophores in cultured hepatocytes as well as work by others, metabolism of these fluorophores in the time frame of our experiments is negligible [48]. Renal and biliary excretion of these fluorophores does occur and for this reason we performed our experiments in a short time frame.

Loss of mitochondrial Rh123 fluorescence by ethanol activation of the MDR transporter was unlikely for several seasons, including 1) identical ethanol-induced changes with Rh123 and TMRM, fluorophores with different membrane permeabilities (Fig. 3) [33]; 2) lack of a characteristic “chicken wire” pattern of biliary excretion of Rh123 that is readily apparent for excreted anionic fluorophores; and 3) no effect of CsA, an MDR inhibitor [34], [35], on Rh123 fluorescence after ethanol treatment. Moreover, 4) mitochondria sequester Rh123 and TMRM dependent on the negative mitochondrial membrane potential, and such sequestered fluorophore is not available for biliary transport. Lastly, 5) mitochondrial depolarization was an all-or-nothing event within individual hepatocytes, and adjacent hepatocytes frequently showed opposite labeling patterns. If ethanol were dose-dependently affecting biliary excretion, we would expect graded responses in all hepatocytes characterized by partial loss of staining at low concentrations and more complete loss at higher concentrations. Moreover, adjacent hepatocytes should respond similarly. Such graded responses were not observed after ethanol. Therefore, we conclude that release of mitochondrial Rh123 and TMRM after ethanol treatment was truly the result of mitochondrial depolarization.

### Ethanol-induced mitochondrial depolarization is not caused by anoxia but is likely due to uncoupling

Alcohol leads to centrolobular hypoxia as determined by pimonidazole staining and direct measurement of oxygen with miniature oxygen electrodes [5], [6], [31], and mitochondrial depolarization could be the consequence of respiratory inhibition due to anoxia. Inconsistent with this hypothesis however, mitochondrial depolarization after acute ethanol treatment was panlobular (Fig. 1), whereas hypoxia only occurs in downstream regions of the liver lobule, namely in centrolobular (pericentral) regions [5],[6],[31]. Since it remains somewhat unclear the degree of hypoxia that is required to achieve pimonidazole immunoreactivity, we imaged the autofluorescence of mitochondrial NAD(P)H simultaneously with mitochondrial polarization status *in vivo* to distinguish anoxia-induced mitochondrial depolarization from that caused by uncoupling. When we imaged mitochondrial NAD(P)H fluorescence at a relatively early time point when some hepatocytes contained polarized mitochondria whereas others were completely depolarized, NAD(P)H fluorescence was decreased only in those hepatocytes with depolarized mitochondria (Fig. 4). Such NAD(P)H oxidation is consistent with mitochondrial uncoupling as the cause of depolarization and inconsistent with anoxia as the cause [49]. Although oxidative stress can cause NAD(P)H oxidation, leading to the MPT onset, depolarization itself after uncoupling also causes NAD(P)H oxidation [29], [30]. Stimulation of respiration by uncoupling can account for increased hepatic oxygen consumption in SIAM and ultimately lead to an increased intralobular oxygen gradient and centrilobular hypoxia. HIF-1α mRNA did not increase at 1 h after ethanol treatment, although mitochondrial depolarization occurred as early as 1 h. HIF-1α showed a trend to increase after 6 h, possibly due to SIAM-induced oxygen consumption consistent with previous reports of HIF-1α activation after longer periods of *in vivo* ethanol treatment [50]. However, under the conditions of our experiments, hypoxia was not severe enough to prevent oxygen-dependent oxidation of NADH by the mitochondrial respiratory chain, which only occurs in the virtual absence of oxygen, namely anoxia. Increased energy expenditure and decreased metabolic efficiency by alcohol have long been noted in human drinkers [51], [52]. Uncoupling provides an explanation for this phenomenon.

### Ethanol metabolism is required for mitochondrial depolarization

The first step of ethanol oxidation forms acetaldehyde, as catalyzed by ADH and to a lesser extent MEOS. In deer mice, ADH deficiency decreased ethanol-induced mitochondrial depolarization by ∼70% compared to wild-types (Fig. 5). Inhibition of MEOS by ABT and deficiency of Cyp2E1, the major subtype of cytochrome-P450 that is responsible for ethanol metabolism by MEOS, decreased mitochondrial depolarization by ∼20% (Fig. 6). Collectively, these data showed that ethanol metabolism was required for mitochondrial depolarization and that ADH plays a larger role than MEOS in producing this phenomenon. This is consistent with the previous observation that 3-methylpyrazole, an ADH inhibitor, strongly blocks the ethanol-induced respiratory burst, whereas ABT blocks to a lesser extent [53]. Other potential pathways (*e.g.*, peroxisomal β-oxidation) might also play a minor role in ethanol metabolism. The effects of combined inhibition of these various pathways on mitochondrial depolarization will be studied in the future.

The second step of ethanol metabolism is oxidation of acetaldehyde to acetate by ALDH in the mitochondrial matrix. Acetaldehyde forms adducts with proteins and is potentially toxic to mitochondria [23]. As measured by MAA adduct formation, ALDH activation decreased whereas ALDH inhibition increased hepatic acetaldehyde concentrations after ethanol treatment (Fig. 7). Likewise, ALDH activation decreased and ALDH inhibition accelerated mitochondrial depolarization after ethanol (Fig. 8). Therefore, acetaldehyde most likely plays an important role in ethanol-induced mitochondrial depolarization, whereas acetate accumulation after acetaldehyde oxidation does not. Interestingly, mitochondria depolarization within individual hepatocytes occurred as an all-or-nothing event rather than a “graded” phenomenon. Partial depolarization was rarely observed. Thus, the acetaldehyde-dependent effect is a threshold phenomenon with different hepatocytes having different thresholds.

Ethanol is readily oxidized to acetaldehyde by isolated hepatocytes, but ethanol caused mitochondrial depolarization *in vivo* but not *in vitro*. Moreover, mitochondrial depolarization occurred at doses of ethanol that exceeded the Km of mouse ADH. Thus, acetaldehyde formation was a necessary step but not the only requirement for onset of mitochondrial depolarization. Other factors existing *in vivo* (*e.g.*, hormonal and neuronal factors, endotoxemia, cross-talk between organs or cells) must also play a role. Our study suggests that increased hepatic oxygen consumption during SIAM is mediated by reversible uncoupling of hepatocellular mitochondria and is consistent with the previous studies which showed that SIAM occurs only after *in vivo* treatment with alcohol and not after *in vitro* treatment of hepatocytes or perfused livers [3], [4], [11]. The mechanism for SIAM remains incompletely understood but most likely involves multiple factors (see **Introduction**) [3], [13], [14]. One or more of these factors may mediate hepatocellular mitochondrial responses to acetaldehyde leading to reversible mitochondrial depolarization.

### Pathological impact of mitochondrial depolarization

Pathological implications of mitochondrial depolarization remain to be established. The mitochondrial uncoupling and respiratory burst after acute ethanol may be, in part, an adaptive response to oxidize toxic acetaldehyde more rapidly and to increase NAD^+^ supply for ADH-dependent alcohol metabolism. Consistent with the reversibility of this mitochondrial depolarization, ALT and apoptosis increased only modestly after acute ethanol, as shown in previous studies [54]–[57], and remained disproportionally low compared to the observed mitochondrial depolarization in 94% of hepatocytes after 6 h (Figs. 2 and 10). Cell death after ethanol was also quite small in comparison to hepatic injury caused by other hepatotoxicants and stresses like acetaminophen and hepatic warm I/R in which ALT increases to >4,000 U/L and histologic necrosis occurs in >40% of hepatocytes [16], [58], [59].

A marked hepatic steatosis occurred after acute ethanol treatment (Fig. 11). Numerous mechanisms may cause steatosis, and conventional wisdom is that increased NADH/NAD^+^ is responsible for steatosis by inhibiting β-oxidation and promoting fatty acid synthesis. However, our results actually show the opposite. Those hepatocytes with depolarized mitochondria and decreased mitochondrial NAD(P)H after ethanol were the ones developing steatosis, whereas adjacent hepatocytes with polarized mitochondrial and high NAD(P)H levels were not steatotic. These results suggest that mitochondrial dysfunction was responsible for hepatic steatosis after acute ethanol (Fig. 11), as is the case for many instances of drug-induced hepatic steatosis [60], [61]. Mitochondrial dysfunction inhibits uptake and oxidation of fatty acyl-CoA, which is instead converted to triglyceride. NADPH is required for *de novo* fatty acid biosynthesis. However, steatosis after acute ethanol was likely not due to increased *de novo* fatty acid biosynthesis regardless of NADPH levels since ATP was decreased (Fig. 10). Rather, ethanol increases adrenergic hormones, which in turn activate adipose hormone-sensitive lipase activity, increasing lipolysis in adipose tissue and causing mobilization of extrahepatic fatty acids that are subsequently transported into the liver. Examination of fatty acid synthesis, lipolysis and NADPH redox state in adipose and liver tissues after ethanol treatment are beyond the scope of this study and will be investigated in the future.

The steady state oxidation-reduction status of mitochondrial pyridine nucleotides in individual hepatocytes reflects the balance of processes reducing and oxidizing mitochondrial NAD(P)H. When ethanol metabolism increases formation of NADH without mitochondrial uncoupling (depolarization) and enhanced respiration, steady state mitochondrial NAD(P)H increases. However, when mitochondrial uncoupling occurs, increased respiration and NADH oxidation ensue that decrease steady state mitochondrial NAD(P)H even in the face of increased NADH formation by ethanol metabolism. Importantly, ALDH activation with Alda-1 to accelerate degradation of acetaldehyde decreased mitochondrial depolarization (Figs. 7 and 8) and also decreased steatosis and measures of liver injury (ALT, TUNEL, ATP depletion) (Figs. 10 and 11). These data are consistent with the conclusion that mitochondrial depolarization caused by acetaldehyde indeed contributes to fat accumulation and liver injury after acute ethanol treatment.

Alcohol affects multiple aspects of lipid metabolism, including increased mobilization of fatty acids from adipose tissue, down-regulation of peroxisome proliferator activated receptor-alpha, decreased hepatic AMP-activated protein kinase activity, activation of sterol regulatory element binding protein-1 and altered adiponectin production [62]–[64]. Plasminogen activator inhibitor-1, HIF-1α, microRNAs, endotoxemia, and the complement system also contribute to ethanol-induced fatty liver [15], [50], [65]–[67]. With progressive liver damage and acute versus chronic alcohol exposure, the predominant mechanisms leading to steatosis may change. Steatosis can increase formation of proinflammatory and profibrogenic cytokines and leptin, which may contribute to development of hepatitis and fibrosis [68], [69]. Alcohol-induced hepatotoxicity is recognized to have a “multi-hit” pathophysiology [70]. Mitochondrial dysfunction may contribute to ALD development as one of these hits. Thus, early hepatic changes like ethanol-induced mitochondrial uncoupling may be important in ALD evolution, leading to fibrosis, cirrhosis and end-stage liver disease.

It is puzzling why acute ethanol causes widespread uncoupling but did not lead to more profound ATP depletion or widespread cell death. A likely explanation is that the liver produces ATP through glycogenolysis and glycolysis, providing an alternative energy source. Our and other studies showed that ATP decreased after ethanol treatment (Fig. 10) [7]–[9]. Such decreased ATP is a powerful stimulus of glycogenolysis, and in confirmation we observed markedly decreased hepatic glycogen after acute ethanol (Fig. 11). Numerous studies have also demonstrated such glycogenolysis after acute ethanol [71], [72]. Although glycolysis provides ATP less efficiently, glycolytic ATP may nonetheless be sufficient to maintain cell survival, as occurs during hepatic ischemia/hypoxia [73].

In cultured hepatocytes, ethanol decreases ATP production without uncoupling [9], [74]. This effect appears related to inhibition of conductance of voltage dependent anion channels (VDAC) in the mitochondrial outer membrane. VDAC is responsible for outer membrane permeability to hydrophilic metabolites like ATP, ADP, fatty acyl-CoA and various respiratory substrates but does not affect entry of membrane-permeant acetaldehyde. In cultured hepatocytes, ethanol and acetaldehyde each inhibit VDAC conductance [74]–[76]. Another consequence of VDAC closure is inhibition of mitochondrial fatty acid oxidation with consequent lipid accumulation and steatosis, since fatty acyl-CoA must pass through VDAC to enter mitochondria for β-oxidation. *In vivo*, mitochondrial uncoupling in combination with VDAC closure promotes more rapid and selective oxidation of acetaldehyde and accounts for the major features of SIAM, as previously proposed [75].

The molecular mechanisms underlying ethanol-induced mitochondrial uncoupling *in vivo* remain to be determined. Opening of cation channels (*e.g.*, mitochondrial calcium uniporter and mitochondrial ATP-sensitive potassium channel), activation/upregulation of uncoupling proteins (*e.g.*, UCP2) and futile calcium cycling across the mitochondrial inner membrane are among several possibilities. However, MPT onset likely does not account for ethanol-induced uncoupling, since mitochondrial depolarization after ethanol was not prevented by CsA, was reversible, did not cause calcein entry into the mitochondrial matrix and did not lead to cell death in the great majority of hepatocytes (Figs. 2,3). Moreover, relatively high acetaldehyde (125 µM) does not inhibit maximal respiratory capacity by rat hepatocytes, indicating that acetaldehyde does not directly inhibit respiratory chain enzymes [74]. Ethanol increased oxidative stress (Fig. 9), and mitochondrial dysfunction can be both a cause and a consequence of generation of reactive oxygen species (ROS) [39], [77]. Future studies will be needed to further elucidate the relation of oxidative stress with mitochondrial depolarization *in vivo*.

Together, our findings using intravital confocal/multiphoton microscopy describe a novel phenomenon of reversible mitochondrial depolarization *in vivo* in mouse livers after ethanol treatment. This phenomenon depends on ethanol metabolism to acetaldehyde mainly by ADH. Mitochondrial depolarization appears due to uncoupling and likely contributes to hepatic steatosis. Depolarization occurs as an all-or-nothing phenomenon in individual hepatocytes, suggestive of a threshold effect. Further work will be needed to elucidate the mechanism of ethanol-induced reversible depolarization, identify the basis of the threshold phenomenon and determine whether individual hepatocytes cycle between polarized and depolarized states.
